# Impact of pH on Regulating Ion Encapsulation of Graphene Oxide Nanoscroll for Pressure Sensing

**DOI:** 10.3390/nano9040548

**Published:** 2019-04-04

**Authors:** Weihao Zhao, Lin Wang, Chengjie Pei, Cong Wei, Hui You, Jindong Zhang, Hai Li

**Affiliations:** Key Laboratory of Flexible Electronics (KLOFE) & Institute of Advanced Materials (IAM), Nanjing Tech University, 30 South Puzhu Road, Nanjing 211816, China; iamwhzhao@njtech.edu.cn (W.Z.); iamwanglin@njtech.edu.cn (L.W.); wojiaopcj@njtech.edu.cn (C.P.); iamcwei@njtech.edu.cn (C.W.); 201861122142@njtech.edu.cn (H.Y.); iamjdzhang@njtech.edu.cn (J.Z.)

**Keywords:** graphene oxide nanoscroll, pH value, ion encapsulation, mesh structure, pressure sensing

## Abstract

Recently, graphene oxide nanoscroll (GONS) has attracted much attention due to its excellent properties. Encapsulation of nanomaterials in GONS can greatly enhance its performance while ion encapsulation is still unexplored. Herein, various ions including hydronium ion (H_3_O^+^), Fe^3+^, Au^3+^, and Zn^2+^ were encapsulated in GONSs by molecular combing acidic graphene oxide (GO) solution. No GONS was obtained when the pH of the GO solution was greater than 9. A few GONSs without encapsulated ion were obtained at the pH of 5–8. When the pH decreased from 5 to 0.15, high-density GONSs with encapsulated ions were formed and the average height of GONS was increased from ~50 to ~190 nm. These results could be attributed to the varied repulsion between carboxylic acid groups located at the edges of GO nanosheets. Encapsulated metal ions were converted to nanoparticles in GONS after high-temperature annealing. The resistance-type device based on reduced GONS (rGONS) mesh with encapsulated H_3_O^+^ showed good response for applied pressure from 600 to 8700 Pa, which manifested much better performance compared with that of a device based on rGONS mesh without H_3_O^+^.

## 1. Introduction

In recent years, graphene and graphene-based composite have exhibited promising pressure sensing behaviors due to their excellent conductivities, remarkable elasticities, and extraordinary stiffness [1,2,3,4,5,6]. Various micro- and nanostructures based on graphene and graphene oxide, including 3D graphene foam [1,2], laser-scribed graphene oxide with crossbar structure [3], graphene oxide-coated polymer mesh membrane, and graphene sponge [4,5,6], have been reported as resistive-type pressure sensors in wearable devices. By rolling up graphene oxide nanosheet, graphene oxide nanoscroll (GONS) is shaped in a spiral form with one-dimensional structure [7,8,9,10,11,12,13,14]. Graphene and graphene oxide nanoscrolls (GONS) have attracted significant attention due to their excellent electrical and mechanical properties, which stem from their opened ends and adjustable interlayer distance [7,8,9,10,11,12,13,14,15,16,17,18,19,20,21,22]. GONS has shown broad applications in the fields of sensors, supercapacitors, field effect transistors, energy storage, and so on [13,14,17,22,23,24,25,26]. Various nanomaterials, such as nanoparticles, nanowires, and small molecules can be encapsulated into GONSs to further improve their performance [13,14,15,19,20,27,28,29,30,31,32,33]. Due to its adjustable interlayer distance, GONS could show resistance variation response to external pressure changes, therefore, it could be predicted as a promising candidate for pressure sensor. Therefore, it is highly desirable to investigate the pressure sensing ability of GONS. 

In this work, we report a simple and reproducible method to encapsulate various ions in GONSs by molecular combing acidic graphene oxide (GO) solution. Due to the strong repulsion between negatively charged carboxylate ions located at the edges of GO nanosheets, no GONS was observed when the pH of GO solution was greater than 9. While a few GONSs were formed on hydrophobic substrate as the pH was in the range of 5–8. The height of GONS prepared from GO solution with pH of 5 (referred to as (GONS)_5_) was ~50 nm, which was kept almost unchanged even after being annealed at 250 °C for 2 h, indicating no hydronium ion (H_3_O^+^) was encapsulated in GONS at this condition. Interestingly, atomic force microscopy (AFM) measurement showed that the height of GONS was increased from ~50 to ~190 nm as the pH value of GO solution was decreased from 5 to 0.15. In addition, the height of GONS prepared from GO solution with pH of 0.15 (referred to as (GONS)_0.15_) was greatly decreased from ~189 to ~72 nm after being annealed at 250 °C for 30 min. The rapidly decreased height of GONS could be attributed to the evaporation of successfully encapsulated H_3_O^+^. Besides H_3_O^+^, other metal ions, including Fe^3+^, Au^3+^, and Zn^2+^ could also be encapsulated into GONSs, which were converted to Fe_x_O_y_, Au, and ZnO nanoparticles in reduced GONSs (rGONS) after being annealed at 480 °C for 30 min. After GO nanosheets were reduced by l -ascorbic acid in solution, H_3_O^+^ could still be encapsulated into rGONS to form the (rGONS)_5_ and (rGONS)_0.3_, respectively. The resistance-type device based on (rGONS)_0.3_ mesh showed excellent response to pressure stimulation compared with that of a device based on (rGONS)_5_ mesh, which could be caused by the enhanced conductivity due to encapsulated H_3_O^+^.

## 2. Materials and Methods

### 2.1. Chemicals and Materials

The main chemicals and materials are listed as follows: Concentrated sulfuric acid (H_2_SO_4_, AR, 98%) was purchased from Wanqing Chemical (Nanjing, China). Iron(III) nitrate nonahydrate (AR, 98.5%), gold chloride trihydrate (AR, 99%), zinc chloride (AR, 99%), toluene (AR, 99%), and trimethoxyoctadecylsilane (OTS, 90%) were purchased from Aladdin (Shanghai, China). Polymethyl methacrylate (PMMA, Mw = 996,000) was purchased from Sigma (Shanghai, China). Polydimethylsiloxane (PDMS) was purchased from Dow Corning (Midland, MI, USA).

### 2.2. Preparation of GO Solution with Various pH

Graphene oxide (GO) was prepared by modified Hummer’s method [34]. KOH and H_2_SO_4_ with appropriate concentration was added to GO solutions to adjust the pH at 10, 9, 8, 7, 6, 5, 4, 3, 2, 1, 0.5, 0.3, and 0. 15, respectively. Before pH adjustment, the GO solution was washed by deionization (DI) water to make the pH 7. In order to make the pH of the GO solution less than 7, small amount of H_2_SO_4_ solution with an appropriate concentration was added to GO solution with pH at 7. To obtain GO solutions with pH at 3, 4, 5, and 6, H_2_SO_4_ solution with a concentration of 10 mM was used to adjust pH. To obtain GO solutions with pH at 0.15, 0.3, 0.5, 1, and 2, H_2_SO_4_ solution with a concentration of 5 M was used to adjust the pH. To obtain GO solutions with pH at 8, 9, and 10, KOH solution with a concentration of 1 mM was used to adjust the pH. To reduce GO in solution, 0.05 g l-ascorbic acid was added in 10 mL GO solution and then kept in the dark environment for 24 h [35,36]. The existence of l-ascorbic acid in GO solution had almost no influence on the formation of rGONS, but served to reduce GO to rGO. It has been reported that GO solution can be stable at ambient conditions for four weeks after reduction with l-ascorbic acid [36]. Therefore, we didn’t wash the l-ascorbic acid out of the solution. To obtain rGO solutions with pH of 5 and 0.3, H_2_SO_4_ solutions with a concentration of 10 mM and 5 M were used to adjust the pH, respectively.

### 2.3. Preparation of GONS and rGONS

To prepare GONS by molecular combing, a hydrophobic substrate is necessary. Firstly, 300 nm SiO_2_/Si substrate was cleaned by piranha solution and then immersed into a glass bottle containing the mixture of 10 mL C_7_H_8_ and 200 μL OTS. After the bottle was heated on a hotplate at 60 °C for 24 h, the SiO_2_/Si substrate was taken out and washed with ethanol and DI water three times each. Thus the hydrophobic OTS–SiO_2_/Si substrate was obtained. Finally, GONS and rGONS was prepared by molecular combing GO and rGO solutions with various pH on the OTS–SiO_2_/Si substrates [13,17,23,26], respectively.

### 2.4. Encapsulation of Nanoparticles into GONS

Fe(NO_3_)_3_, HAuCl_4_, and ZnCl_2_ were first dissolved into GO solution with a pH of 0.3 at a concentration of 50 mM each. Next, metal ions (Fe^3+^, Au^3+^, and Zn^2+^) were encapsulated into GONSs by the molecular combing method. After the GONSs with metal ions were heated at 480 °C for 30 min, the Fe_x_O_y_, Au, and ZnO nanoparticles were obtained in rGONS, respectively. 

### 2.5. Fabrication of a Device Based on rGONS Mesh and Pressure Sensing Test

After rGONS was prepared by molecular combing, a rGONS mesh with size up to several millimeters was fabricated between two PDMS films by using our previously reported transfer method [13,26,37]. The resistance-type device based on rGONS mesh was fabricated by depositing conductive Cu tapes on the diagonal corners as two electrodes. Pressure sensing test was performed on a semiconductor characterization system (Keithley 4200). 

### 2.6. Characterization

An optical microscope (Axio Scope A1, Zeiss, Oberkochen, Germany) was used to image the GONS and related samples. Atomic force microscopy (Dimension ICON, Bruker, Santa Barbara, CA, USA) was employed to measure the height of GONS and GONS with encapsulated nanomaterials. In order to image the evaporation process of H_3_O^+^, GONSs were heated at 100, 150, 200, 250, and 300 °C for 30 min, respectively. In order to continually monitor the evaporation process, GONSs were heated at 250 °C for 30, 60, 90, and 120 min, respectively. Transmission electron microscope (JEOL 2100F) was used to characterize the rGONS–iron oxide nanoparticles (Fe_x_O_y_ NPs).

## 3. Results and Discussion

### 3.1. Effect of pH on Encapsulating H_3_O^+^ in GONS

**Figure 1:**
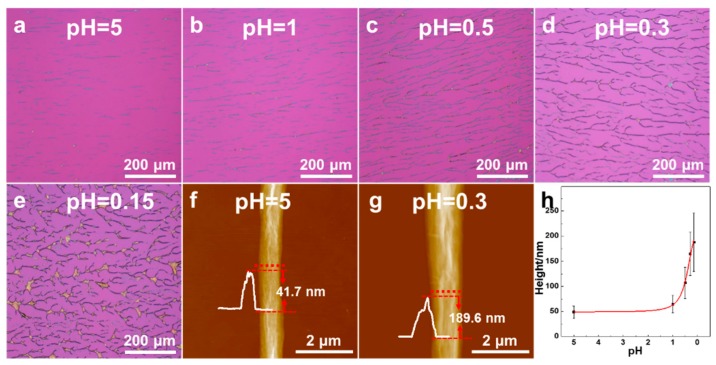
(**a**–**e**) Optical microscopy images of a graphene oxide nanoscroll (GONS) prepared by molecular combing graphene oxide (GO) solutions with a pH of 5 (**a**), 1 (**b**), 0.5 (**c**), 0.3 (**d**), and 0.15 (**e**), respectively. (**f**,**g**) Atomic force microscopy (AFM) images of GONSs prepared by molecular combing GO solutions with pH of 5 (**f**) and 0.3 (**g**), respectively. (**h**) The height plot of GONS as the function of solution pH.

As previously reported, GONS can be prepared by molecular combing GO solution on hydrophobic substrates [13,17,23,26]. Herein, GONSs with various heights were prepared by molecular combing GO solution with a controlled pH. Figure 1a–e shows the optical microscopy (OM) images of GONSs deposited on OTS–SiO_2_/Si substrates by molecular combing GO solutions with pH of 5, 1, 0.5, 0.3, and 0.15, respectively. As-prepared GONSs are named as (GONS)_5_, (GONS)_1_, (GONS)_0.5_, (GONS)_0.3_, and (GONS)_0.15_, respectively. As shown in Figure 1a, straight and light blue (GONS)_5_ lines with a length of several tens of µm were observed. AFM measurement showed the height of (GONS)_5_ was 41.7 nm (Figure 1f), which is consistent with previously reported results [12,17]. As the pH of the GO solution was decreased to 1 (Figure 1b), parallel and light blue (GONS)_1_ lines with length from several tens to hundreds of micrometers were observed. While dense parallel (GONS)_0.5_ lines with length of hundreds of micrometers were deposited on the OTS–SiO_2_/Si substrate, as shown in Figure 1c. Meanwhile, some (GONS)_0.5_ lines showed black color, indicating their heights are larger than those of light blue (GONS)_0.5_ lines (Figure S1 in Supporting Information (SI)). As the pH was decreased to 0.3, black (GONS)_0.3_ lines with length of hundreds of micrometers and typical height of 189.6 nm were prepared (Figure 1d,g). Moreover, dendric (GONS)_0.3_ lines were formed at this condition. As the pH of the GO solution was further decreased to 0.15, more wide black dendric (GONS)_0.15_ lines were observed in Figure 1e, compared to the (GONS)_0.3_ lines shown in Figure 1d. In addition, some irregular big joints with khaki color were observed, which could be acid droplets trapped in GO nanosheets due to the high concentration of H_3_O^+^. As shown in Figure 1h, the height of the GONS was rapidly increased from ~50 to ~190 nm as the pH was decreased from 5 to 0.15. Since the GO nanosheets used in these experiments were synthesized from the same batch, the size of GO nanosheets should have negligible influence on the formation of GONS. Thus the pH of the GO solution should play an important role in determining the height and morphology of GONS, that is, the encapsulation of H_3_O^+^.

### 3.2. De-Encapsulation of H_3_O^+^ from GONS at High Temperature

In order to investigate the stability of encapsulated H_3_O^+^ in GONS, as-prepared GONSs shown in Figure 1 were heated at a high temperature for some time. Firstly, (GONS)_5_ was continually heated at 100, 150, 200, 250, and 300 °C for 30 min, respectively. AFM measurement indicated that its height was almost kept unchanged after high-temperature annealing (Figure S2a in SI). This behavior implies two possibilities. One is the encapsulated H_3_O^+^ was quite stable even at 300 °C for 30 min, and the other possible reason is that there was no encapsulated H_3_O^+^ in (GONS)_5_. In order to explore the conceivable reason, (GONS)_1_, (GONS)_0.5_, and (GONS)_0.3_ were also annealed at the same condition (Figure S2b–d in SI). As measured by AFM, the height of (GONS)_1_ was slightly decreased from ~83 to ~80 nm when it was heated at 100 °C for 30 min. The height of (GONS)_1_ was continually decreased from ~80 to ~67 nm when it was further annealed at 150 °C for 30 min (Figure S2b in SI), which could be attributed to the evaporation of encapsulated H_3_O^+^ at high temperature. In order to confirm the evaporation, (GONS)_1_ was annealed at 200 °C for another 30 min. As measured by AFM, the height of (GONS)_1_ was greatly decreased from ~67 to ~32 nm (Figure S2b in SI). The rapid height decrease of (GONS)_1_ could confirm the evaporation of encapsulated H_3_O^+^ in (GONS)_1_ at high temperature. Therefore, no H_3_O^+^ was encapsulated in (GONS)_5_, due to the maintained height after high-temperature annealing. When the annealing temperature was increased to 250 and 300 °C, the height of (GONS)_1_ decreased slightly from 32 to 30 nm and 26 nm after 30 min annealing, respectively, indicating most of the encapsulated H_3_O^+^ was evaporated at 200 °C. Similar rapid height decrease was also observed in (GONS)_0.5_ and (GONS)_0.3_ at the same conditions (Figure S2c,d in SI), respectively.

**Figure 2:**
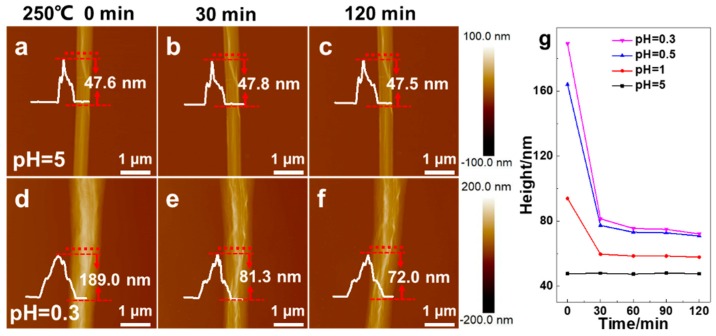
(**a**–**c**) AFM images of (GONS)_5_ (**a**) before and after being annealed at 250 °C for 30 (**b**) and 120 (**c**) min, respectively. (**d**–**f**) AFM images of (GONS)_0.3_ (**d**) before and after being annealed at 250 °C for 30 (**e**), and 120 (**f**) min, respectively. (**g**) The height plots of GONSs prepared at pH of 0.3, 0.5, 1, and 5 as a function of annealing time at 250 °C, respectively.

Figure 2 shows the AFM characterization on the evaporation of H_3_O^+^ in (GONS)_5_ and (GONS)_0.3_ when they were continually heated at 250 °C for 30, 60, 90, and 120 min, respectively. As shown in Figure 2a–c,g, the height of (GONS)_5_ was maintained at around 47 nm even after 120 min annealing at 250 °C, confirming no H_3_O^+^ was encapsulated in (GONS)_5_. The tiny height difference within 0.2 nm could come from the measurement variation instead of evaporation of H_3_O^+^. However, the height of (GONS)_0.3_ was obviously decreased from 189.0 to 81.3 nm after 30 min annealing at 250 °C (Figure 2d,e), indicating a large amount of H_3_O^+^ was evaporated. The height of (GONS)_0.3_ was slightly decreased from 81.3 to 72.0 nm after additional 90 min annealing at 250 °C (Figure 2f), indicating the encapsulated H_3_O^+^ was quickly evaporated at the first stage of annealing. As shown in Figure 2g, similar phenomena were also observed when (GONS)_1_ and (GONS)_0.5_ were annealed at 250 °C, respectively. The aforementioned results indicated that more H_3_O^+^ was encapsulated in (GONS)_0.5_ and (GONS)_0.3_ compared to that in (GONS)_1_, further confirming the important role of pH in encapsulating H_3_O^+^.

### 3.3. Encapsulation of Other Ions in GONS

**Figure 3:**
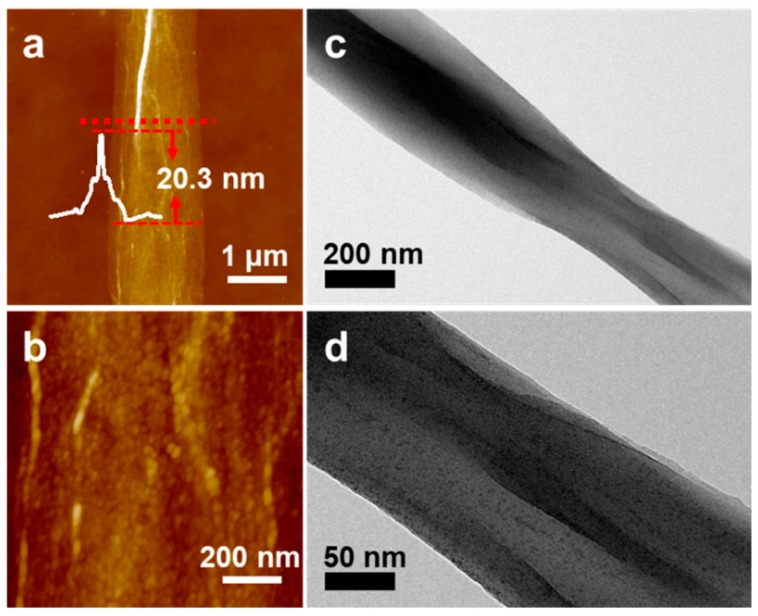
(**a**,**b**) AFM height and (**c**,**d**) TEM images of (rGONS-Fe_x_O_y_ NP)_0.3_.

Besides H_3_O^+^, other ions can also be encapsulated in GONS. Firstly, Fe(NO_3_)_3_ was dissolved in GO solution (GO–Fe) at a concentration of 0.05 M and the pH of the solution was adjusted to 0.3. GO–Fe nanoscrolls were fabricated by molecular combing, which was named as (GONS-Fe)_0.3_. As measured by AFM, the height of (GONS-Fe)_0.3_ was 153.3 nm (Figure S3a in SI). In addition, the (GONS-Fe)_0.3_ has a smooth surface, which is similar to that of (GONS)_0.3_. After the (GONS-Fe)_0.3_ was annealed at 480 °C for 30 min, high-density iron oxide nanoparticles (Fe_x_O_y_ NPs) were clearly observed in nanoscroll (Figure 3b), indicating the Fe(NO_3_)_3_ was converted to Fe_x_O_y_ NPs. Meanwhile, the GONS was reduced to rGONS at high temperature and thus (rGONS-Fe_x_O_y_ NP)_0.3_ was obtained. As shown in Figure 3a, the height of (rGONS-Fe_x_O_y_ NP)_0.3_ was 20.3 nm, which is much lower than that of (GONS-Fe)_0.3_. The decreased height should be attributed to the evaporation of encapsulated H_3_O^+^ and partial etching of GO at 480 °C. As shown in the TEM images of (rGONS-Fe_x_O_y_ NP)_0.3_ (Figure 3c,d), nanoparticles can be clearly observed. Similarly, Au and ZnO nanoparticles were also encapsulated in GONS by this way (Figure S3 in SI), indicating the versatility of our method.

### 3.4. Pressure Sensing Performance of the Device Based on rGONS Mesh

**Figure 4:**
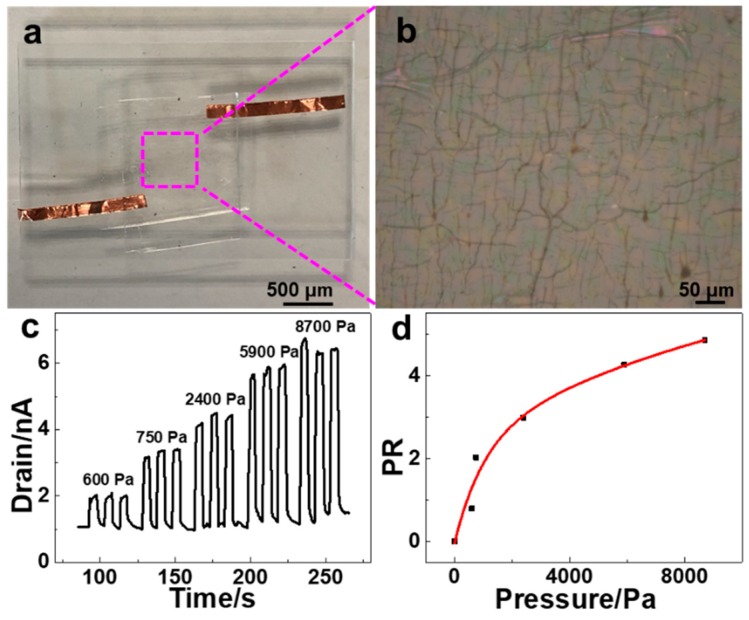
(**a**) Photograph and (**b**) optical microscopy image of pressure sensor based on (rGONS)_0.3_ mesh. (**c**) Time-dependent current response plot of the (rGONS)_0.3_ mesh-based device at different pressures. (**d**) Pressure response plot of the (rGONS)_0.3_ mesh-based device as a function of applied pressure.

Previously, we have reported that rGONS mesh can be used as a flexible electrode and humidity sensor but the interlayer distance of rGONS cannot be modulated [13,14,26]. Since the interlayer distance of GONS can be greatly enlarged by encapsulating H_3_O^+^, GONSs could act as potential materials for pressure sensing. In order to reduce the GO in solution to enhance its conductivity, l-ascorbic acid was used [35,36]. Interestingly, rGO nanoscrolls with encapsulated H_3_O^+^ can also be fabricated by molecular combing rGO solution with various pH (Figure S4 in SI). Subsequently, (rGONS)_0.3_ mesh was fabricated by cross aligning (rGONS)_0.3_ lines as previously reported [26]. After the resistance-type device based on (rGONS)_0.3_ mesh was prepared (Figure 4a,b), its pressure sensing performance was tested. As shown in Figure 4c, the device showed good response when pressures of 600, 750, 2400, 5900, and 8700 Pa were applied on the device at a drain voltage of 1 V, respectively. In order to describe the pressure sensing performance, pressure response (PR) of the device was expressed as follows,

PR = (I_P_ − I_0_)/I_0_

Here, the I_P_ is the measured current of the device with applied pressure, and the I_0_ is the measured current of the device without pressure, respectively.

As shown in Figure 4d, the PR of (rGONS)_0.3_ mesh device showed rapid increase when pressure less than 750 Pa was applied. While slight increase was observed with further increased pressure, indicating the device is sensitive to lower pressure. We have measured the conductivities of (rGONS)_5_ and (rGONS)_0.3_ mesh devices. As shown in Figure S5a in SI, the conductivity of the (rGONS)_0.3_ mesh device is much higher than that of the (rGONS)_5_ mesh device. At a drain voltage of 5 V, the current of the (rGONS)_0.3_ mesh device was around 6 nA while that of (rGONS)_5_ mesh device was only around 5 pA. The current of the (rGONS)_5_ mesh device showed good linear relation to the drain voltage at 0–5 V, as shown in Figure S5b in SI, indicating it is conductive. Compared to the excellent response of the (rGONS)_0.3_ mesh device shown in Figure 4c,d, the (rGONS)_5_ mesh device showed instable and insensitive response for pressure less than 1000 Pa. Figure S5c in SI shows the response of the (rGONS)_5_ mesh device at a pressure of 2400 Pa and drain voltage of 1 V. Although the (rGONS)_5_ mesh device showed better sensitivity in this case, the signal was quite instable.

### 3.5. Discussion of the pH Regulation on the Encapsulation of Ions in GONS

**Scheme 1:**
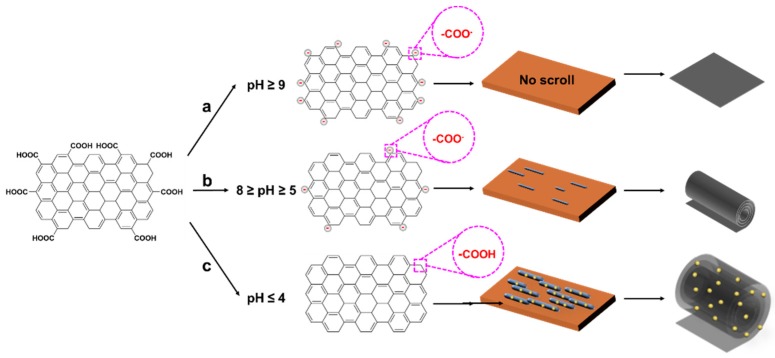
Schematic diagram of H_3_O^+^ encapsulation in GONS at different pH.

It is well accepted that graphene oxide has various functional groups, including epoxide and hydroxyl groups on the basal plane as well as carboxylic acid groups located at the edges [38,39]. When the pH of the GO solution is greater than 9, the carboxylic acid groups could become to negatively charged carboxylate ions. Due to the strong repulsion between the negatively charged carboxylate ions, self-rolling up of GO cannot be started from the edges. Therefore, no GONS is formed at this condition, as illustrated by the Route a in Scheme 1. If the pH of the GO solution is in the range of 5–8, partially negatively charged carboxylate ions are located at the edges. In this case, the repulsion between negatively charged ions is decreased and a few GONSs are formed, as illustrated by Route b in Scheme 1. When the pH of the GO solution is lower than 4, neutral carboxylic acid groups are located at the edges and there is no repulsion between them. Thus high-density GONSs are deposited on the hydrophobic substrate during molecular combing operation, as shown by Route c in Scheme 1. Moreover, many H_3_O^+^ ions are also encapsulated into GONS to enlarge the height (diameter) of GONS. These proposed explanations are consistent with the experimental results shown in Figure 1 and Figure S6 in SI. As shown in Figure S6a–v, there were a few GONSs in OM images when graphene oxide (GO) solutions with pH less than 8 were used for molecular combing. However, when a GO solution with pH of 9 was used for molecular combing, there was no GONS observed in the OM images (Figure S6w,x). Similar phenomenon was also observed for a GO solution with pH of 10 (Figure S6y,z). Moreover, when GO was reduced to rGO by l-ascorbic acid, only the epoxide and hydroxyl groups on the basal plane were removed [35]. The carboxylic acid groups were still maintained at the edges. Therefore, the pH regulation on the height (diameter) and high yield of rGONS were also observed simultaneously (Figures S4 and S7 in SI), further confirming that our proposed mechanism is feasible.

## 4. Conclusions

In summary, various ions, including H_3_O^+^, Fe^3+^, Au^3+^, and Zn^2+^, were successfully encapsulated in GONS by molecular combing acidic GO solution with corresponding ions. The concentration of H_3_O^+^ (pH) played an important role in encapsulating ions in GONS. When the pH of the GO solution was higher than 9, no GONS was prepared due to the strong repulsion between negatively charged carboxylate ions located at the edges of the GO. A few GONSs were obtained when the pH of GO solution was in the range of 5–8. As the pH of GO solution decreased from 5 to 0.15, the height of GONS was increased from ~50 to ~190 nm due to increased interlayer distance by encapsulating H_3_O^+^. The embedded H_3_O^+^ in GONS can be completely removed by annealing at 300 °C for 120 min. By dissolving metal ions into a GO solution with pH of 0.3, GONSs with encapsulated metal ions were prepared. As-encapsulated metal ions were converted to metal or metal oxide nanoparticles when they were annealed at 480 °C for 30 min. By reducing GO to rGO with l-ascorbic acid in solution, H_3_O^+^ embedded rGONS can be fabricated by molecular combing rGO solution with a pH of 0.3. The resistance-type device based on the (rGONS)_0.3_ mesh showed good response to applied pressure on it compared with that of device based on (rGONS)_5_ mesh without H_3_O^+^. Our results indicated that the pH of GO solution not only played an important role in regulating the ion encapsulation of GONS but also enhanced the pressure sensing performance of rGONS mesh-based device due to the increased interlayer distance. Our study not only provides an alternative method to encapsulate functional nanomaterials in GONS, but also reveals the formation mechanism of GONS.

## Supplementary Materials

The following are available online at https://www.mdpi.com/2079-4991/9/4/548/s1, **Figure S1:** Optical image of (GONS)_0.5_ lines with black and light blue color. **Top inset:** AFM image of (GONS)_0.5_ line with black color. **Bottom inset:** AFM image of (GONS)_0.5_ line with light blue color. **Figure S2:** Plots of heights of (**a**) (GONS)_5_, (**b**) (GONS)_1_, (**c**) (GONS)_0.5_, and (**d**) (GONS)_0.3_ as a function of 30 min annealing at temperature of 100, 150, 200, 250, and 300 °C, respectively. **Figure S3:** AFM height images of GONSs containing (**a**) Fe^3+^, (**b**) Au^3+^, and (**c**) Zn^2+^ before annealing, respectively. AFM height images of rGONSs containing (**d**) FexOy NPs, (**e**) Au NPs, and (**f**) ZnO NPs after annealing at 480 °C for 30 min, respectively. (**g–i**) Magnified AFM images of (**d–f**), respectively. **Figure S4:** Optical images of (**a**) (rGONS)_0.5_, (**b**) (rGONS)_0.3_, and (**c**) (rGONS)_0.15_, respectively. **Figure S5:** (**a**) The I–V plots of (rGONS)_5_ and (rGONS)_0.3_ mesh devices at drain voltage of 0–5 V. (**b**) Magnified plot of the black curve shown in (**a**). (**c**) Pressure response of (rGONS)_5_ mesh device at a pressure of 2400 Pa. **Figure S6:** Optical images of GONSs prepared at pH of 0.15 (**a,b**), 0.3 (**c,d**), 0.5 (**e,f**), 1 (**g,h**), 2 (**i,j**), 3 (**k,l**), 4 (**m,n**), 5 (**o,p**), 6 (**q,r**), 7 (**s,t**), 8 (**u,v**), 9 (**w,x**), and 10 (**y,z**), respectively. **Figure S7:** AFM height images of (**a**) (GONS)_0.3_ and (**b**) (rGONS)_0.3_, respectively.
