# Supplementary material for: Impact of pH on Regulating Ion Encapsulation of Graphene Oxide Nanoscroll for Pressure Sensing
Source: Nanomaterials (Basel). 2019 Apr 4;9(4):548. doi: 10.3390/nano9040548 (PMC6523837; doi:10.3390/nano9040548)
Supplement: Supplementary file 1 [file nanomaterials-09-00548-s001.pdf]

nanomaterials

MDPI

Article

Supporting Information

Impact of pH on Regulating Ions Encapsulation of Graphene Oxide Nanoscroll for Pressure Sensing

Weihao Zhao, Lin Wang, Chengjie Pei, Cong Wei, Hui You, Jindong Zhang and Hai Li *

Key Laboratory of Flexible Electronics (KLOFE) & Institute of Advanced Materials (IAM), Nanjing Tech University, 30 South Puzhu Road, Nanjing 211816, China; iamwhzhao@njtech.edu.cn (W.Z.); iamwanglin@njtech.edu.cn (L.W.); wojiaopcj@njtech.edu.cn (C.P.); iamcwei@njtech.edu.cn (C.W.); 201861122142@njtech.edu.cn (H.Y.); iamjdzhang@njtech.edu.cn (J.Z.)

* Correspondence: iamhli@njtech.edu.cn

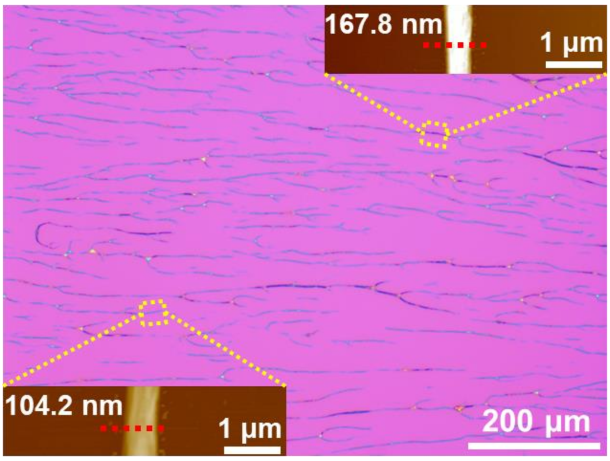

Figure S1. Optical image of $(GONS)_{0.5}$ lines with black and light blue color. Top inset: AFM image of $(GONS)_{0.5}$ line with black color. Bottom inset: AFM image of $(GONS)_{0.5}$ line with light blue color.

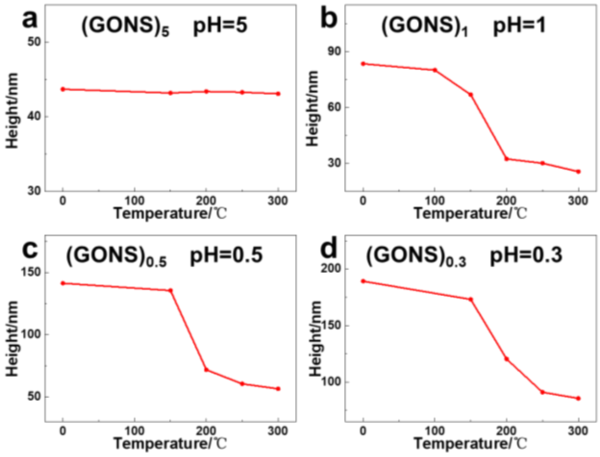

Figure S2. Plots of heights of (a) $(GONS)_5$, (b) $(GONS)_1$, (c) $(GONS)_{0.5}$ and (d) $(GONS)_{0.3}$ as a function of 30 min annealing at temperature of 100 ℃, 150 ℃, 200 ℃, 250 ℃ and 300 ℃, respectively.

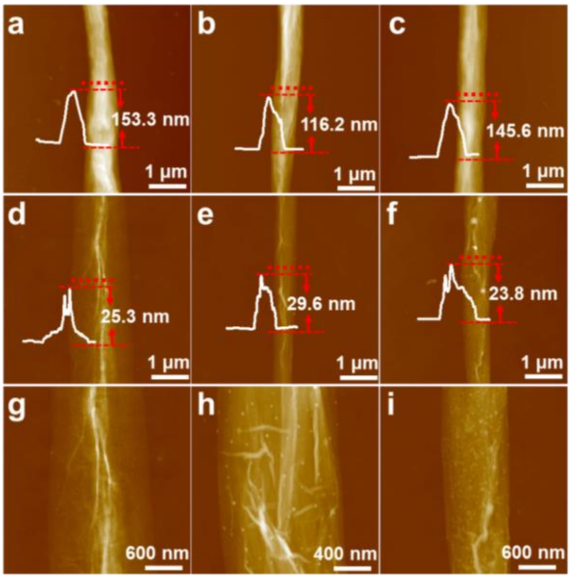

Figure S3. AFM height images of GONSs containing (a) Fe^{3+}, (b) Au^{3+} and (c) Zn^{2+} before annealing, respectively. AFM height images of rGONSs containing (d) FexOy NPs, (e) Au NPs and (f) ZnO NPs after annealing at 480 ℃ for 30 min, respectively. (g-i) Magnified AFM images of (d-f), respectively.

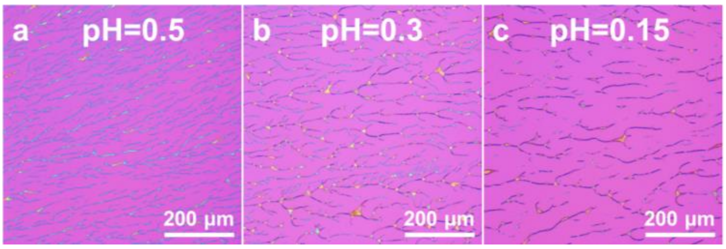

Figure S4. Optical images of (a) $(rGONS)_{0.5}$, (b) $(rGONS)_{0.3}$ and (c) $(rGONS)_{0.15}$, respectively.

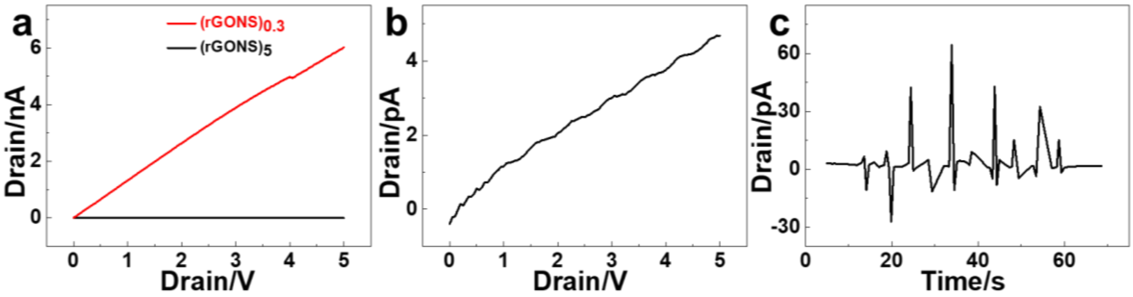

Figure S5. (a) The I-V plots of $(rGONS)_5$ and $(rGONS)_{0.3}$ mesh devices at drain voltage of 0-5 V. (b) Magnified plot of the black curve shown in (a). (c) Pressure response of $(rGONS)_5$ mesh device at a pressure of 2400 Pa.

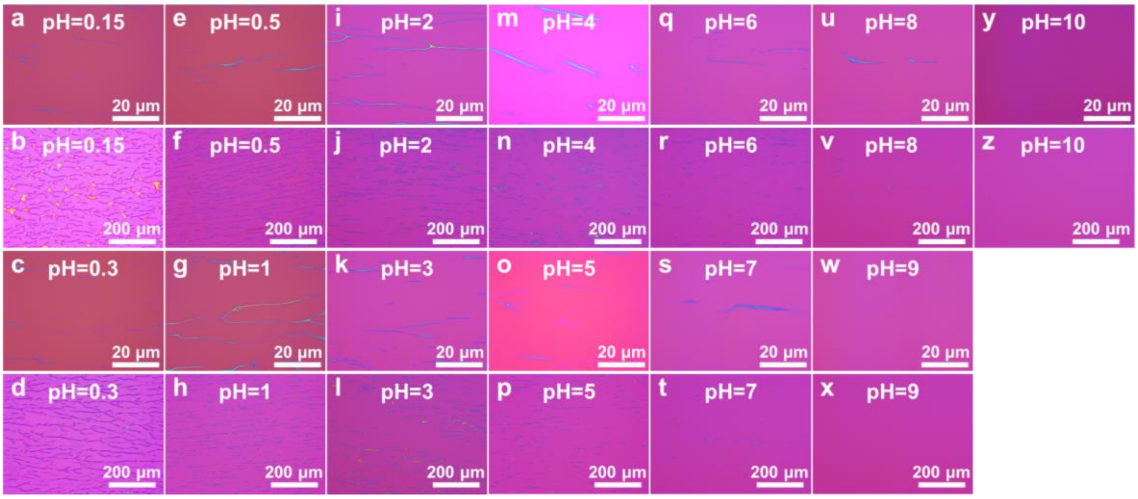

Figure S6. Optical images of GONSs prepared at pH of 0.15 (a-b), 0.3 (c-d), 0.5 (e-f), 1 (g-h), 2 (i-j), 3 (k-l), 4 (m-n), 5 (o-p), 6 (q-r), 7 (s-t), 8 (u-v), 9 (w-x) and 10 (y-z), respectively.

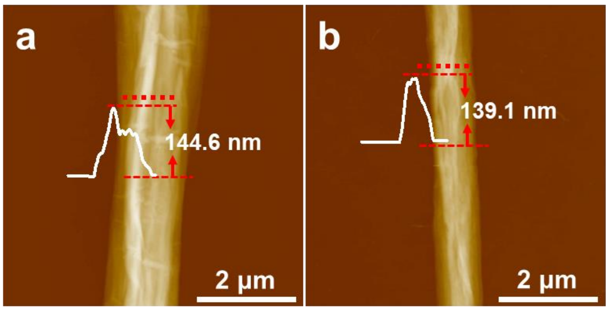

Figure S7. AFM height images of (a) $(GONS)_{0.3}$ and (b) $(rGONS)_{0.3}$, respectively.

© 2018 by the authors. Submitted for possible open access publication under the terms and conditions of the Creative Commons Attribution (CC BY) license (http://creativecommons.org/licenses/by/4.0/).
